# ^211^At targeted alpha therapy in Europe: overcoming clinical, regulatory, and infrastructure challenges

**DOI:** 10.1007/s00259-026-07950-y

**Published:** 2026-05-29

**Authors:** Hugo Levillain, Anne Royer Moës, Cristiana Gameiro-Paris, Antero Abrunhosa, Tom Deakin, Geraldine Gebhart, Matthias M. Herth, Jean-François Gestin, Ferid Haddad, Andreas Ingemann Jensen, Rebecca Lo Bue, Renata Mikolajczak, Adrian Otamendi, Katie Staunton-Mann, Kevin Tabury, Patrick Flamen

**Affiliations:** 1 Institut Jules Bordet, Université Libre de Bruxelles (ULB), Hôpital Universitaire de Bruxelles (H.U.B), Brussels, Belgium; 2 IBA SA, RadioPharmaSolutions, Brussels, Belgium; 3 Institute for Nuclear Sciences Applied to Health (ICNAS), University of Coimbra, Coimbra, Portugal; 4 LabLogic Systems Limited, Sheffield, UK; 5 TetraKit Technologies ApS, University of Copenhagen, Copenhagen, Denmark; 6 Department of Drug Design and Pharmacology, University of Copenhagen, Copenhagen, Denmark; 7 Nantes Université, Inserm, CNRS, Université d’Angers, CRCI2NA Nantes, France; 8 GIP Arronax, Saint-Herblain, and Subatech, Nantes, France; 9 The Oncidium Foundation, Brussels, Belgium; 10 Radioisotope Centre POLATOM, National Centre for Nuclear Research, Otwock, Poland; 11 Vicomtech, San Sebastian, Spain; 12 Belgian Nuclear Research Centre (SCK CEN), Mol, Belgium

**Keywords:** Astatine-211, Targeted alpha therapy, Radiopharmacy, Self-imaging, Theranostics, ACCELERATE, Clinical trials

## Abstract

Targeted Alpha Therapy (TAT) holds considerable promise for precision oncology, yet its clinical deployment in Europe remains limited. Beyond scientific considerations, progress is constrained by fragmentation across the radiopharmaceutical value chain, encompassing radionuclide production, radiochemistry, preclinical validation, clinical development, regulatory frameworks, infrastructure, and workforce training. This editorial focuses on astatine‑211 (^211^At) as a strategically important radionuclide for European targeted alpha therapy. Its favorable decay characteristics, chemical versatility, and theranostic potential make ^211^At a strong candidate for clinical translation. However, despite advances in radiochemistry and encouraging early‑phase clinical experience, current evidence remains largely preclinical or restricted to first‑in‑human studies, and broader clinical adoption remains uncertain. We argue that the main barriers are not isolated technical obstacles but a self‑reinforcing dynamic linking limited supply, restricted clinical evidence generation, regulatory complexity, and insufficient workforce readiness. Addressing these challenges sequentially or in isolation is unlikely to succeed. Instead, coordinated, value‑chain‑driven approaches are required, in which production, preclinical research, clinical evaluation, regulation, and training progress together. From a European perspective, initiatives such as ACCELERATE.EU: an EU-HORIZON-JU-IHI funded project bringing together a consortium of 17 partners across Europe with expertise in ^211^At and targeted alpha therapies, illustrate how distributed production networks, harmonized practices, co‑clinical development, and structured workforce training can contribute to greater autonomy, resilience, and equitable patient access. Establishing a decentralized and sustainable European supply model for ^211^At should therefore be considered a strategic priority to enable multicenter clinical trials, supply independence and support the future integration of targeted alpha therapy into routine clinical practice.

## A new momentum for targeted alpha therapy

Targeted Alpha Therapy (TAT) delivers high‑linear energy transfer (LET) radiation capable of eradicating tumor cells, including micrometastatic and treatment‑resistant disease, while largely sparing surrounding healthy tissues [1, 2]. Despite this strong radiobiological rationale, clinical adoption of TAT has remained limited, largely due to the restricted availability of alpha‑emitting radionuclides combining suitable decay properties, feasible chemistry, and scalable production.

Several alpha‑emitters have been explored for therapeutic use, including ^223^Ra, ^225^Ac, ^213^Bi, ^212^Pb, and ^212^Bi, each associated with distinct physical and radiochemical constraints. Within this context and supported by a European COST program (CA11914/www.astatine-net), astatine‑211 (^211^At) has emerged as a particularly promising candidate, owing to its short half‑life of 7.2 h, relatively simple decay scheme, and halogen chemistry compatible with established iodine‑inspired radiolabeling strategies [3–5].

However, growing scientific interest has not been matched by production capacity. Global availability of alpha‑emitting radionuclides remains limited, creating a major bottleneck for broader clinical evaluation and adoption. Recent analyses have highlighted substantial international disparities and emphasized the need for coordinated European infrastructure to secure sustainable supply chains for ^211^At and other alpha emitters [6]. At present, ^211^At production relies on medium‑energy cyclotrons (~ 30 MeV) equipped with alpha‑beam capabilities, restricting access to a small number of specialized research centers. As a result, a central question persists: can ^211^At overcome production, regulatory, and logistical hurdles to become a routinely used clinical tool [3]?

Beyond these individual constraints, the translation of ^211^At‑based targeted alpha therapy is further impeded by a self‑reinforcing “chicken‑and‑egg” dynamic, in which limited and unreliable supply restricts clinical evidence generation; insufficient evidence fails to generate the level of confidence required to trigger sustained investment; and delayed investment, in turn, constrains the development of production capacity, infrastructure, and workforce training. Breaking this deadlock cannot be achieved through incremental or sequential actions. It requires deliberate, coordinated strategies in which production, new radiochemistry strategies, supply, preclinical innovation, clinical evaluation, regulation, and training are advanced hand-in-hand as interdependent components of a single value‑chain effort.

European initiatives such as ACCELERATE.EU aim to break this cycle by building coordinated, resilient, and autonomous frameworks that federate existing production capacities for immediate research needs while simultaneously preparing the transition toward scalable manufacturing and harmonized production and clinical implementation, with the objective of enabling equitable patient access to ^211^At‑based therapies and strengthening Europe’s leadership in targeted alpha therapy.

## Why ^211^At deserves attention

### Radiophysical rationale

**Fig. 1:**
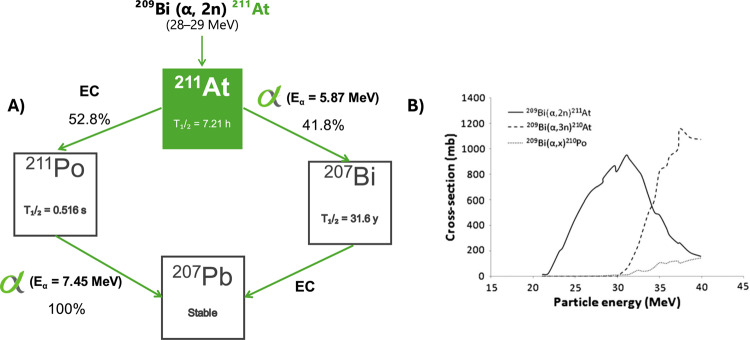
**A** Nuclear reaction for production of ^**211**^**At** from ^**2**^**⁰⁹Bi** and decay scheme, **B** Cross section of the irradiation of.^**2**^**⁰⁹Bi** by α particles, Reproduced and adapted, with permission from Guérard et al., 2013

^211^At decays via alpha emission, delivering a single high-energy alpha particle per disintegration (See Fig. 1). Its short tissue penetration (~ 50–80 µm) allows highly localized cytotoxicity while sparing surrounding tissue, and the simple decay scheme may simplify dosimetry evaluation [1].

Beyond its therapeutic properties, the radioactive decay of ^211^At gives rise to detectable secondary emissions that have motivated the exploration of X‑ray–based self‑imaging approaches, although their clinical readiness remains limited. The decay of ^211^At to ^211^Po is accompanied by low‑energy X‑ray emissions (77–92 keV) that can, in principle, be detected using novel ring‑shaped CZT‑based gamma‑camera systems equipped with tungsten rather than lead collimators. The absence of lead eliminates interference from lead X‑ray fluorescence, allowing the astatine‑related emissions (which appear as a broadened composite peak due to limited energy resolution) to be identified more effectively, thereby enabling self‑imaging and early assessment of tumor targeting. In this context, such approaches are conceptually aligned with recent regulatory guidance emphasizing the importance of early biodistribution and activity verification for therapeutic radiopharmaceuticals [7–9]. Nevertheless, the proximity of tungsten X‑ray peak poses challenges for optimal energy window definition and scatter estimation, indicating that further technical optimization and validation are required. Accordingly, X‑ray‑based “self‑imaging” remains technically demanding and is currently most relevant in controlled preclinical or early clinical research settings, although recent proof‑of‑concept work, notably from Japanese groups, has demonstrated feasibility at the level of image acquisition, without yet establishing quantitative or clinically actionable performance.

Chemical similarity with iodine and fluorine supports the exploration of halogen‑based surrogate strategies for ^211^At‑labelled radiopharmaceuticals. Iodine‑based pairs such as ^211^At/^123^I and, in more exploratory settings, ^124^I are conceptually well suited for investigating biodistribution, pharmacokinetics, and, in selected cases, dosimetric relationships. However, despite its theoretical relevance, the practical use of ^124^I remains challenging in Europe due to production, regulatory, and logistical constraints. By contrast, short‑lived halogen PET tracers such as ^1^⁸F may offer complementary information primarily for patient selection, but are less suited for extrapolating biodistribution or dosimetry. Importantly, differences in radiochemistry, in vivo stability, and pharmacokinetics necessitate careful validation, and direct dosimetric extrapolation to ^211^At‑based therapies cannot be assumed a priori.

Short‑lived PET isotopes such as ⁶⁸Ga may also be explored primarily for qualitative target assessment or patient selection. As illustrated by established theranostic paradigms such as PSMA‑ and somatostatin‑receptor–based radiopharmaceuticals, differences in radionuclide half‑life, emitted radiation, and even molecular structure do not preclude clinical utility for selection purposes. However, in the context of ^211^At‑based therapy, such PET surrogates should be regarded primarily as exploratory tools for patient selection rather than for predictive dosimetry or direct clinical surrogacy [5, 10].

### Radiochemical uniqueness and stability

In radiopharmaceutical applications, ^211^At behaves predominantly as a halogen. Its position as the heaviest halogen supports iodine-inspired chemistry, while its increased polarizability and partial metallic character enable alternative bonding strategies. A major historical limitation has been the in vivo instability of astatine-labelled compounds due to deastatination, often attributed to the weakness and polarity of the At–C bond and to oxidative transformations of astatine in biological environments.

Recent radiochemistry developments have therefore focused on strengthening astatine incorporation at the molecular level. In particular, the use of electron-rich aromatic systems (e.g., aryl stannane or boronic precursors) has improved electrophilic substitution efficiency, while the development of more robust At–C(sp^2^) bonds and the introduction of At–B bonds (e.g., closo-borate scaffolds) have demonstrated enhanced resistance to in vivo cleavage. These approaches reduce the release of free astatine and improve biological stability compared to earlier alkyl-based linkages [4, 5, 11, 12].

In parallel, a better understanding of astatine redox chemistry, especially the control of At⁻/At⁰/At⁺ species through appropriate oxidizing agents and stabilizing conditions, has enabled more reproducible labeling, formulation, and handling at clinically relevant activity levels [13, 14]. However, despite these advances, challenges remain in fully controlling astatine speciation and ensuring long-term in vivo stability across different molecular vectors.

### Biological advantage

High-LET, short-range alpha emissions are ideal for targeting micrometastases, minimal residual disease, and disseminated tumor cells while minimizing damage to adjacent healthy tissues. Preclinical studies across solid tumors and hematologic malignancies have demonstrated robust tumor control and prolonged survival benefit [1, 8, 15–17].

Targeted alpha therapy has been shown, in preclinical radiobiology studies, to exert potent direct cytotoxic effects on tumor cells and, in addition, to induce forms of tumor cell death associated with immunogenic features, including the release of damage‑associated molecular patterns and enhanced antigen presentation. These observations have led to the hypothesis that alpha‑particle irradiation may promote immunogenic cell death and potentially synergize with immunotherapy by remodeling the tumor microenvironment [1, 18]. In addition, the cytotoxic mechanism of alpha particles is largely independent of tumor oxygenation and cell cycle status, rendering targeted alpha therapy effective against hypoxic, quiescent, or radioresistant cells, while also disrupting tumor vasculature and hypoxic niches without inducing systemic toxicity [5, 19].

However, it should be emphasized that evidence supporting immunogenic cell death and synergy with immunotherapies remains largely preclinical, indirect and is primarily derived from general radiobiological studies rather than from experimental or clinical data specific to ^211^At‑based radiopharmaceuticals. At present, direct demonstrations of clinically meaningful synergy between targeted alpha therapy and immune checkpoint inhibitors or other immunotherapies remain limited.

Accordingly, the potential immunomodulatory effects of ^211^At‑based therapies should be regarded as an emerging concept, warranting further mechanistic investigation and prospective evaluation in dedicated preclinical models and clinical studies, rather than as an established or clinically validated feature.

### Clinical potential and ongoing trials

Clinical development of ^211^At‑based radiopharmaceuticals is currently centered on early‑phase clinical trials, primarily designed to assess safety, dosimetry, and feasibility rather than therapeutic efficacy. As summarized in Table 1, ongoing studies explore a diverse range of molecular targets and clinical indications, reflecting the broad potential applicability of ^211^At‑based targeted alpha therapy [16, 20].

**Table 1:** Overview of ongoing early‑phase clinical trials with ^211^At‑based radiopharmaceuticals

Trial ID	Radiopharmaceutical/Target	Indication	Phase	Primary objective	Sponsor
NCT04083183	^211^At-BC8-B10 (anti-CD45)	Non-malignant diseases (conditioning)	Phase 1/2	Safety, feasibility	Fred Hutchinson Cancer Center
NCT04579523	^211^At-OKT10-B10 (anti-CD38)	Multiple myeloma	Phase 1	Safety, conditioning	Fred Hutchinson Cancer Center
NCT05275946	[^211^At]NaAt	Differentiated thyroid cancer	Phase 1	Safety, dosimetry	Osaka University
NCT06441994	[^211^At]PSMA-5	Castration-resistant prostate cancer	Phase 1	Safety, PK, dosimetry	Osaka University/Alpha Fusion
NCT07287748	AF-001-NaAt	Differentiated thyroid cancer	Phase 1a/1b	Safety, dose escalation	Alpha Fusion
NCT07260162	ATO-101™ (girentuximab)	Non-muscle-invasive bladder cancer	Phase 1	Safety, tolerability	Atonco/ICO

Current clinical efforts encompass small‑molecule ligands (e.g. PSMA‑ and sodium astatide–based approaches) as well as antibody‑based and radioimmunoconjugate strategies targeting hematologic and solid malignancies, including differentiated thyroid cancer, prostate cancer, bladder cancer, and hematologic disorders. Across these programs, Phase I and Phase I/II studies focus on dose escalation, biodistribution, and tolerability, with efficacy‑related endpoints remaining exploratory.

In parallel, numerous programs are emerging from newly created biotechnology companies, including Naya Therapeutics, Z‑Alpha, NTx Therapeutics, and Iodax, while many additional candidates are advancing through late preclinical development. Collectively, this expanding pipeline highlights growing interest in ^211^At‑based therapeutics. However, while early clinical signals are encouraging, conclusions regarding clinical efficacy remain premature and will require confirmation in larger, later‑phase trials.

## From promise to practice: the challenges

### Production and supply

^211^At is produced via ^209^Bi(α,2n)^211^At, requiring medium-energy alpha-beam cyclotrons [21, 22]. ^211^At can be isolated via either dry distillation or wet chemistry processes, with modern optimized solid-phase extraction methods providing high recovery yields (> 90%) and effective separation of ^211^At from ^210^At and ^210^Po impurities [5]. Worldwide and European availability is limited; only a handful of research centres currently operate the required α-beam cyclotrons. In [6], a total of 15 facilities producing ^211^At have been identified. In Europe [23], current producing site are Rigshospitalet (Denmark) with an end of bombardment (EOB) batch size ∼3 GBq, GIP Arronax (France) with an EOB batch size of ∼2 GBq and FZJ (Germany) with an EOB batch size of ∼ 5.6 GBq. Depending on the facility, the number of batch per year vary from 50 to 200 and has increased steeply over the last years. Other facilities are about to start (NCBJ in Poland) or to restart (Rez, Czechia and Birmingham, UK). Finally, one center has indicated their interest to start ^211^At production (Jyväskylä, Finland). In parallel to this academic network, several private companies are working to setup production networks such as Alpha Nuclide (Zhejiang, China), or IONETIX (USA) or Framatome/IBA (Europe). These networks are based on dedicated cyclotron that are, for some of them, designed to optimize ^211^At production [6]. In parallel, NOAR Europe and the World Astatine Community (WAC) have been created to further enhance scientific research, expand global availability of ^211^At, and stimulate clinical interest. Collectively, these initiatives are expected to increase production capacity and availability in the coming years.

However, determining what constitutes “sufficient” supply cannot be dissociated from the requirements of the downstream value chain. Within ACCELERATE.EU, the production network is deliberately coupled to the parallel development of multiple radiopharmaceutical candidates at different stages, including purely preclinical programs (NTR1 for PDAC), early‑phase clinical trials (^211^At‑Substance P for GBM), and hybrid pipelines spanning preclinical and Phase I evaluation (^211^At‑DualFAPi for TNBC). This integrated setup allows production requirements to be continuously challenged and refined based on real development needs, rather than fixed assumptions. In this context, production capacity is not merely a supply issue, but an adaptive parameter that must co‑evolve with preclinical validation, clinical trial design, and regulatory constraints.

### Radiochemistry challenges

Astatine’s multiple oxidation states (–1, 0, + 1, + 3, + 5), sensitivity to solvent and redox conditions, and susceptibility to α‑induced radiolysis pose specific technical challenges in ^211^At processing [4, 5, 12, 24–27]. These include:

1. achieving reproducible speciation control at tracer levels,
2. preventing radiolytic oxidation or reduction during high‑activity processing,
3. managing volatility under certain conditions
4. ensuring consistent stability of At-vector (At-C, At-B, At-M), which remains highly dependent on precursor design and conjugate structure.

Current mitigation strategies rely on controlled redox environments, radical scavengers, and rapid automated syntheses, although full control of astatine speciation remains elusive.

Post‑irradiation processing introduces additional sources of variability. Depending on the production site, ^211^At can be isolated using dry‑distillation or wet‑chemistry dissolution workflows, which differ in operational robustness, impurity profiles, and downstream compatibility [24]. Recent studies have refined wet‑chemistry approaches, particularly through optimized solid‑phase extraction, to improve ^211^At recovery and to better characterize ^21^⁰At and its decay‑related ^21^⁰Po contaminants, thereby enhancing reproducibility and radiochemical purity [28].

Progress has also been made in improving bond stability through optimized molecular scaffolds, including aryl/alkyl, silicon/metal‑based, and boron‑cluster systems, which show increased resistance to in vivo deastatination. However, stability remains highly structure‑dependent. Radiolysis and oxidative processes during labeling can still reduce incorporation efficiency and compromise reproducibility, despite the use of stabilizing additives and optimized solvent systems, while astatine volatility under oxidizing conditions requires careful control of chemical form during processing.

In vivo, residual deastatination may lead to the release of free ^211^At and subsequent uptake in the thyroid and stomach due to halogen affinity, and has also been observed to a lesser extent in other organs such as the spleen and lungs. This effect can be mitigated through standard iodine‑blocking protocols, which substantially reduce thyroid and gastric uptake and render it manageable rather than a fundamental limitation, even though limited residual distribution may still occur in other tissues depending on astatine speciation [10, 29]. Taken together, these considerations indicate that astatine radiochemistry is currently characterized by progressive mitigation strategies rather than complete chemical control, with ongoing advances improving reliability but not yet eliminating fundamental constraints.

### Regulatory considerations

EU Clinical Trial Regulation (EU No. 536/2014) requires Good Manufacturing Practices (GMP) compliance for investigational medicinal products. Early-phase trials may use non-GMP radionuclides even if the final radiopharmaceutical is GMP-compliant [30]. Production practices need to be standardized for future industrial production.

### Transport and logistics

Transport of radioactive material is governed by IAEA SSR-6, ADR, RID, and IATA Dangerous Goods Regulations [7, 31]. Ongoing discussions on the adaptation of transport frameworks for therapeutic alpha‑emitters further highlight the importance of production strategies compatible with short‑lived radionuclides. Compliance with these regulations is essential for ^211^At distribution and reinforces the need for near‑site production or rapid courier networks.

### Workforce and training

The safe and reproducible production and clinical implementation of ^211^At‑based radiopharmaceuticals require highly specialized multidisciplinary teams, including cyclotron engineers, radiochemists, GMP radiopharmacists, quality control specialists, medical physicists, radiation safety officers, and trained clinical staff. While a limited number of European research centers have developed strong expertise in ^211^At production and handling, this expertise remains concentrated, and opportunities for broader workforce training, particularly on the clinical side, remain limited.

Similar challenges have been observed during the clinical implementation of therapeutic radionuclides more broadly. In Europe, structured training and workforce development have primarily emerged around beta‑emitting therapies such as ^131^I and, more recently, ^1^⁷⁷Lu, reflecting their broad integration into routine clinical practice and reimbursement frameworks. Other beta‑emitters, including ⁹⁰Y, have been used in more specialized settings (e.g. radioembolization or radioimmunotherapy), but have not driven comparable, widespread workforce structuring across nuclear medicine. By contrast, alpha‑emitting therapies, including ^225^Ac‑, ^212^Pb‑, and ^211^At‑based approaches, remain largely confined to research and early‑phase clinical studies, which restricts opportunities for hands‑on clinical training and limits the expansion of experienced multidisciplinary teams.

To address this gap, ACCELERATE.EU explicitly integrates workforce development as a core component of its clinical and infrastructural strategy. Our effort is coordinated with other European players such as other EU funded projects like PRISMAP and PRISMAP + or professional associations like the EANM, the IAEA and NOAR Europe. In addition to securing isotope supply, the initiative supports the development of dedicated educational and training activities, including harmonized curricula, standardized training materials, and hands‑on modules covering cyclotron operation, radiochemistry, GMP radiopharmacy, quality control, transport, and clinical administration. Cross‑center mobility schemes and structured knowledge exchange between participating institutions further contribute to consistent skill development across Europe.

The relatively short half‑life of ^211^At (7.2 h) necessitates relative proximity between production sites and clinical use, inherently favoring a distributed production model across multiple countries and institutions. This structural constraint increases the demand for qualified personnel at several sites simultaneously and reinforces the need for harmonized training frameworks and interoperable procedures. In a European context characterized by cross‑border collaboration and personnel mobility, consistent training standards are essential to ensure safe operations, regulatory compliance, and reproducibility when staff move between production and clinical sites. By linking workforce training directly to coordinated multicenter early‑phase clinical studies, ACCELERATE.EU enables multidisciplinary teams to acquire practical experience within real‑world clinical environments. Through this integrated approach, workforce development is no longer treated as an aspirational objective but as a foundational operational component of a sustainable European ecosystem for ^211^At‑based targeted alpha therapy.

### Infrastructure needs for scaling clinical deployment of ^211^At

Only a few European centers currently produce clinical-grade ^211^At; the Alpha Atlas documents this scarcity [6]. To up-scale access, Europe needs:

- Additional medium-energy α-beam cyclotrons and coordinated production networks;
- Shielded hot cells and containment handling;
- Automated synthesis modules;
- GMP-compliant clean rooms, qualified QC labs capable of gamma-spectrometry and radio-chromatography techniques.
- Secure treatment facility able to safely administer alpha-therapy cycles, including waste handling [32]
- Logistics solutions respecting SSR-6/ADR/RID/IATA constraints [31, 33, 34].

## Call to action: from fragmented development to coordinated value‑chain acceleration

In our view, this situation reflects a broader systemic deadlock across the radiopharmaceutical value chain. As illustrated throughout this editorial, progress cannot be unlocked through isolated or step‑by‑step actions. Meaningful advancement requires coordinated and interoperable frameworks in which production, clinical development, regulation, and workforce training are deliberately aligned and advanced hand‑in‑hand.

Breaking silos is central to this approach. Multidisciplinary collaboration does not merely bring different experts together, it enables continuous mutual adaptation across the entire value chain. When production specialists, radiochemists, preclinical scientists, clinicians, regulators, and training experts work in close interaction, constraints and practical limitations are shared early, solutions can be co‑designed, and development choices adjusted in real time. This integrated way of working helps ensure that regulatory requirements and workforce needs are anticipated rather than addressed late, avoiding fragmentation that can otherwise hinder multicenter trials, scalable deployment, and equitable access. As a result, development timelines can be shortened and the risk of non‑actionable or non‑translatable solutions substantially reduced.

Within this landscape, initiatives such as ACCELERATE.EU illustrate how a coordinated value‑chain approach can be implemented in practice. By federating existing European production capacities to provide predictable access to ^211^At for research and early clinical studies, while simultaneously preparing the transition toward scalable and industrial‑grade production through dedicated alpha‑cyclotron development, the initiative deliberately connects short‑term feasibility with long‑term clinical ambition. In parallel, it integrates ligand development, radiolabelling optimization, preclinical and co‑clinical research, early‑phase clinical trials, regulatory alignment, and workforce training as interdependent elements rather than independent tasks.

Importantly, ACCELERATE.EU should be viewed as a demonstrator rather than a unique solution. The rationale reflected in its name is the conviction that accelerating patient access requires replacing siloed development with coordinated, multidisciplinary collaboration across the entire value chain. While specific technical challenges will differ between radionuclides and targeting vectors, the need for early alignment between supply, clinical evidence generation, regulatory readiness, and workforce capacity is common to radiopharmaceutical therapy as a whole.

Ultimately, the successful and sustainable integration of ^211^At‑based therapies, and radiotheranostics more broadly, will depend on whether the community continues to address challenges separately, or instead commits to coherent, value‑chain‑driven strategies that align scientific innovation with clinical, regulatory, and operational reality. Europe has the expertise, infrastructure, and collaborative capacity to lead this transition. What is now required is a shared commitment to work together across disciplines and borders, so that promising radiopharmaceuticals can move more efficiently from concept to meaningful patient benefit.

## Data Availability

NA.
